# Prognostic factors for the management of chondral defects of the knee and ankle joint: a systematic review

**DOI:** 10.1007/s00068-022-02155-y

**Published:** 2022-11-07

**Authors:** Filippo Migliorini, Nicola Maffulli, Jörg Eschweiler, Christian Götze, Frank Hildebrand, Marcel Betsch

**Affiliations:** 1 Department of Orthopaedic, Trauma and Reconstructive Surgery, RWTH University Hospital Aachen, 52074 Aachen, Germany; 2 Department of Orthopaedic and Trauma Surgery, Eifelklinik St. Brigida, 52152 Simmerath, Germany; 3 Department of Medicine, Surgery and Dentistry, University of Salerno, 84081 Baronissi, Italy; 4 School of Pharmacy and Bioengineering, Faculty of Medicine, Keele University, Thornburrow Drive, Stoke on Trent, England; 5 Barts and the London School of Medicine and Dentistry, Centre for Sports and Exercise Medicine, Queen Mary University of London, Mile End Hospital, London, E1 4DG England; 6 Department of Orthopaedics, Auguste-Viktoria-Klinik, 32545 Bad Oeynhausen, Germany; 7 Department of Orthopaedics and Trauma Surgery, University Hospital Erlangen, 91054 Erlangen, Germany

**Keywords:** Chondral defect, Knee, Talus, Prognostic factors

## Abstract

**Purpose:**

Different surgical techniques to manage cartilage defects are available, including microfracture (MFx), autologous chondrocyte implantation (ACI), osteoarticular auto- or allograft transplantation (OAT), autologous matrix-induced chondrogenesis (AMIC). This study investigated the patient-related prognostic factors on the clinical outcomes of surgically treated knee and ankle cartilage defects.

**Methods:**

This study followed the PRISMA statement. In May 2022, the following databases were accessed: PubMed, Google Scholar, Embase, and Scopus. All the studies investigating the outcomes of surgical management for knee and/or talus chondral defects were accessed. Only studies performing mesenchymal stem cells transplantation, OAT, MFx, ACI, and AMIC were considered. A multiple linear model regression analysis through the Pearson Product–Moment Correlation Coefficient was used.

**Results:**

Data from 184 articles (8905 procedures) were retrieved. Female sex showed a positive moderate association with visual analogue scale at last follow-up (*P* = 0.02). Patient age had a negative association with the American Orthopaedic Foot and Ankle Score (*P* = 0.04) and Lysholm Knee Scoring Scale (*P* = 0.03). BMI was strongly associated with graft hypertrophy (*P* = 0.01). Greater values of VAS at baseline negatively correlate with lower values of Tegner Activity Scale at last follow-up (*P* < 0.0001).

**Conclusion:**

The clinical outcomes were mostly related to the patients’ performance status prior surgery. A greater BMI was associated with greater rate of hypertrophy. Female sex and older age evidenced fair influence, while symptom duration prior to the surgical intervention and cartilage defect size evidenced no association with the surgical outcome. Lesion size and symptom duration did not evidence any association with the surgical outcome.

**Supplementary Information:**

The online version contains supplementary material available at 10.1007/s00068-022-02155-y.

## Introduction

The treatment of articular cartilage defects of the lower extremities is challenging given the poor recruitment of regenerative cells into the defect, which results in a limited self-healing capacity of native cartilage tissue [1, 2]. Patients with full-thickness cartilage defects may experience significant pain, decrease in joint function and quality of life [3]. Cartilage defects result from trauma or repetitive shear and torsional forces applied to the cartilage surface [4]. If left untreated, cartilage lesions can progress to early osteoarthritis, joint pain, and mobility impairment [5, 6]. The previous studies estimated that the incidence of full-thickness cartilage lesions varies from 5% to 10% in knees of patients undergoing arthroscopy [7–9]. The prevalence of cartilage lesions in the ankle joint varies from 40% to 95% in patients with persistent pain after an ankle sprain with and without chronic ankle instability [10].

Microfracture (MFX) is considered the first-line treatment for cartilage defects, in both the knee and ankle joints, given the simplicity of the procedure, its low cost, minimal invasiveness, and satisfactory short-term clinical outcome [11, 12]. However, there have been concerns regarding the durability of MFX over time, since the clinical outcomes may worsen over time, in particular in larger lesions and more active patients [13–15]. To overcome limitations of the MFX technique, various forms of autologous chondrocyte implantation (ACI) and osteoarticular transfer system (OATS) have been introduced [16]. Although multiple techniques have been suggested to be effective in the management of articular cartilage defects, international recommendations and clear guidelines are missing. Moreover, it is also uncertain in which patient-dependent factors predict success after any of the above-mentioned cartilage treatment options.

Therefore, a systematic review was conducted to investigate whether patient characteristics at baseline exert an influence on surgical outcome in terms of patient-reported outcome reports (PROMs) and complications.

## Materials and methods

### Search strategy and data source

This systematic review is according to the Preferred Reporting Items for Systematic Reviews and Meta-Analyses: the PRISMA statement [17]. The literature search was conducted independently by two authors (F.M. & J.E.). In May 2022, the following databases were accessed: PubMed, Google scholar, Embase, and Scopus. The following keywords were used in combination: *chondral*, *cartilage*, *articular*, *damage*, *defect*, *injury*, *chondropathy*, *knee*, *pain*, *matrix-induced*, *periosteal*, *periosteum*, *collagen*, *autologous*, *chondrocyte*, *transplantation*, *implantation*, *MFX*, *microfractures*, *mosaicplasty*, *mACI*, *cACI*, *pACI*, *AMIC*, *OAT*, *osteochondral transplantation*, *allograft*, *autograft*, *membrane*, *therapy*, *management*, *surgery*, *outcomes*, *revision*, *hypertrophy*, *failure.* Articles resulting from the literature search were screened by the same authors. The full text of the articles of interest was accessed. The bibliographies were also screened by hand for inclusion. Disagreements were solved by a third author (NM).

### Eligibility criteria

All the studies investigating the outcomes of surgical management for knee and/or talus chondral defects were accessed. Only studies performing OAT, MFX, ACI or AMIC were considered. Given the authors language abilities, articles in English, German, Italian, French and Spanish were eligible. Studies with level I to IV of evidence, according to Oxford Centre of Evidence-Based Medicine [18], were considered. Abstracts, reviews, comments, editorial and opinion were not considered. Animals, biomechanics or in vitro studies were not considered. Studies enhancing the surgical procedures with stem cells were also eligible. Only studies that clearly stated the nature of the surgical intervention were included. Studies which included patients with large chondral and/or osteochondral lesions (>5 cm^2^) and obese (BMI > 30 kg/m^2^) were not included. Studies that reported data on patients with end-stage joint degeneration were not eligible. Only articles reporting quantitative data under the outcomes of interest were considered for inclusion. Missing data under the outcomes of interest warranted the exclusion from this study.

### Outcomes of interest

Data extraction was performed separately by two authors (F.M. & J.E.). Data concerning author, year, journal, type of study and length of the follow-up were collected. The following data at baseline were collected: the number of patients, age, sex, mean BMI (kg/m^2^), size of the defect (cm^2^), and duration of symptoms (months). Data concerning the following scores were extracted at baseline and at last follow-up: visual analogic scale (VAS), American Orthopedic Foot and Ankle Score (AOFAS) [19], Tegner Activity Scale [20], Lysholm Knee Scoring Scale [21], and International Knee Documentation Committee (IKDC) [22] scores. Furthermore, rate of hypertrophy, failures, and revisions were also retrieved. The primary outcome was to investigate the association of baseline patient-specific characteristics on surgical outcomes following restorative cartilage procedures for the knee and ankle.

### Methodology quality assessment

The methodological quality assessment was performed by two authors independently (F.M. & J.E.). The risk of bias graph tool of the Review Manager Software (The Nordic Cochrane Collaboration, Copenhagen) was used. The following risk of bias was evaluated: selection, detection, attrition, and other source of bias.

### Statistical analysis

All statistical analyses were performed by one author (F.M.) using the software STATA/MP 14.1 (StataCorp, College Station, TX). The Shapiro–Wilk test was performed to investigate data distribution. For normal data, mean and standard deviation were calculated. For nonparametric data, median and interquartile range were calculated. The Student’s *T* test was used to assess significance for parametric data, while the Mann–Whitney *U*-test was used for nonparametric variables. Values of *P* < 0.05 considered statistically significant. A multivariate analysis was performed to assess associations between data of patients at baseline with the clinical scores at last follow-up and complications. A multiple linear model regression analysis through the Pearson Product–Moment Correlation Coefficient ($r$) was used. The Cauchy–Schwarz formula was used for inequality: + 1 is considered as positive linear association, while − 1 a negative one. Values of 0.1 <|$r$ |< 0.3, 0.3 <|$r$ |< 0.5, and |$r$ |> 0.5 were considered to have weak, moderate, and strong association, respectively. The overall assessment of significance was performed using the χ^2^ test, with values of *P* < 0.05 considered statistically significant.

## Results

### Search result

**Fig. 1:**
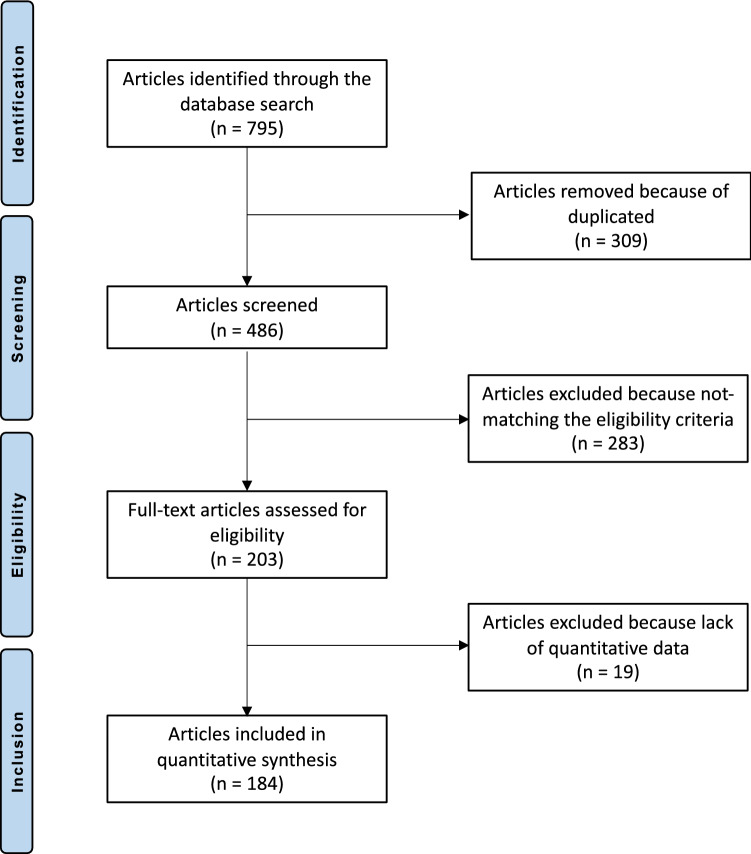
Flowchart of the literature search

The literature search resulted in 795 articles. Of them, 309 were duplicates. A further 302 articles were not eligible: surgical technique (*N* = 74), not focusing on knee or ankle (*N* = 41) study design (*N* = 140), not reporting quantitative data under the outcomes of interest (*N* = 19), other (*N* = 24), language limitations (*N* = 4). This left 184 articles for the present study. The literature search results are shown in Fig. 1.

### Methodological quality assessment

**Fig. 2:**
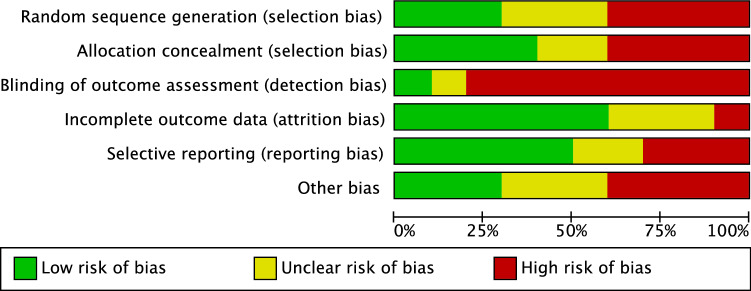
Methodological quality assessment

The risk of selection bias was judged as moderate. Indeed, 46.7% of studies (86 of 184) performed a retrospective analysis, while 38.0% (70 of 184) were prospective, and 15.2% (28 of 184) were randomized. Only few studies (35 of 184) performed assessor blinding; thus, the risk of detection bias was high. The risk of attrition and reporting bias were moderate, as was the risk of other bias. In conclusion, the overall review authors' judgements about each risk of bias item scored moderate, attesting to this study acceptable methodological assessment. The risk of bias graph is shown in Fig. 2.

### Patient demographics

Data from 8905 procedures were retrieved. The median duration of symptoms before the index surgery was 36 (23.6–50.8) months. 41.7% (3713 of 8905) of patients were women. The mean age of the patients was 33.9 ± 6.9 years, while the mean BMI 25.6 ± 1.5 kg/m^2^. The mean defect size was 3.3 ± 2.3 cm^2^. The median follow-up time was 41.8 (24 to 60) months. Generalities and demographic data of the study are shown in Table 1.

**Table 1:** Generalities and patient baseline of the included studies

Author, year	Journal	Study Design	Follow-up (*months*)	Place	Type of treatment	Procedures (*n*)	Female (*%*)	Mean age
Adams et al. 2011 [42]	*J Bone Joint Surg*	Retrospective	48.0	Talus	OAT	8	62.5%	31.4
Adams et al. 2018 [43]	*Foot Ankle Int*	Prospective	55.0	Talus	OAT	14	42.9%	40.0
Ahmad et al. 2015 [44]	*Foot Ankle Int*	Randomized	40.5	Talus	OAT	16	37.5%	39.7
			35.2	Talus	OAT	20	45.0%	41.3
Akgun et al. 2015 [45]	*Arch Orthop Trauma Surg*	Prospective, randomized	24.0	Knee	Syn-MSC	7	57.1%	32.3
				Knee	mACI	7	57.1%	32.7
Albano et al. 2017 [46]	*BMC Musculos Dis*	Retrospective	30.0	Talus	AMIC	16	50.0%	42.6
Anders et al. 2012 [47]	*Int Orthop*	Prospective	63.5	Talus	MACI	22	22.7%	23.9
Anders et al. 2013 [48]	*Open Orthop J*	Prospective, randomized	24.0	Knee	AMIC	8	12.0%	35.0
				Knee	AMIC	13	23.0%	39.0
				Knee	MFX	6	33.0%	41.0
Apprich et al. 2012 [49]	*Osteoarthritis Cartilage*	Retrospective	48.0	Talus	MACT	10	60.0%	31.0
			59.6	Talus	MFX	10	40.0%	32.4
Astur et al. 2018 [50]	*Rev Bras Orthop*	Prospective	12.0	Knee	AMIC	7	14.3%	37.2
Aurich et al. 2010 [51]	*Am J Sports Med*	Retrospective	24.5	Talus	MACI	19	27.8%	29.2
Bartlett et al. 2005 [52]	*J Bone Joint Surg*	Prospective, randomized	12.0	Knee	cACI	44	40.7%	33.7
				Knee	mACI	47		33.4
Basad et al. 2010 [53]	*Knee Surg Sports Traumatol Arthrosc*	Prospective, randomized	24.0	Knee	mACI	40	38.0%	33.0
				Knee	MFX	20	15.0%	37.5
Basad et al. 2015 [54]	*Knee Surg Sports Traumatol Arthrosc*	Prospective	60.0	Knee	mACI	25	37.0%	32.0
Battaglia et al. 2011 [55]	*Knee Surg Sports Traumatol Arthrosc*	Retrospective	60.0	Talus	MACI	20	30.0%	35.0
Baumfeld et al. 2018 [56]	*Foot*	Retrospective	10.8	Talus	AMIC	17	47.1%	37.5
Baums et al. 2007 [57]	*J Bone Joint Surg*	Retrospective	63.0	Talus	PACI	12	58.3%	29.7
Becher et al. 2015 [58]	*Arch Orthop Trauma Surg*	Prospective	21.0	Knee	MFX	5	40.0%	27.0
Becher et al. 2017 [59]	*J Orthop Surg Res*	Prospective, randomized	36.0	Knee	mACI	25	32.0%	33.0
				Knee	mACI	25	16.0%	34.0
				Knee	mACI	25	40.0%	34.0
Becher et al. 2018 [60]	*Knee Surg Sports Traumatol Arthrosc*	Retrospective	67.2	Talus	MFX	16	56.3%	33.3
			68.4	Talus	AMIC	16	56.3%	32.4
Behrens et al. 2006 [61]	*Knee*	Prospective	34.5	Knee	mACI	38	50.0%	35.0
Bentley et al. 2012 [62]	*J Bone Joint Surg*	Prospective, randomized	120.0	Knee	pACI, cACI	58	43.1%	31.0
				Knee	Mosaicplasty	42	59.5%	32.0
Berruto et al. 2017 [63]	*Injury*	Prospective	162.0	Knee	pACI	9	31.3%	31.6
				Knee	cACI	24		
Bode et al. 2013 [64]	*Arch Orthop Trauma Surg*	Prospective	71.9	Knee	cACI	19		40.2
				Knee	cACI	24		38.3
Brittberg et al. 2018 [65]	*Am J Sports Med*	Prospective, randomized	60.0	Knee	mACI	65	38.0%	35.0
				Knee	MFX	63	33.0%	34.0
Browne et al. 2006 [66]	*Clin Orthop Rel Res*	Prospective	60.0	Knee	pACI	100	35.0%	37.0
Buda et al. 2010 [67]	*J Bone Joint Surg*	Prospective	29.0	Knee	BM- MSC	20	40.0%	
Buda et al. 2015 [68]	*Int Orthop*	Retrospective	48.0	Talus	MACI	40	37.5%	31.4
				Talus	BMC	40	32.5%	30.2
Buda et al. 2019 [69]	*Europ J Orthop Trauma Surg*	Retrospective	48.0	Knee	BMAC	28	42.9%	38.0
Chan et al. 2018 [70]	*Cartilage*	Prospective	65.8	Talus	pACI	24	41.7%	34.1
Chung et al. 2014 [71]	*Knee Surg Sports Traumatol Arthrosc*	Prospective	24.0	Knee	MFX	12	83.3%	44.3
				Knee	AMIC	24	41.7%	47.4
Cole et al. 2011 [72]	*Am J Sports Med*	Prospective, randomized	24.0	Knee	Control group			
				Knee	MFX	9	44.0%	33.0
Cole et al. 2012 [72]	*Am J Sports Med*	Prospective	48.0	Knee	pACI	32	25.0%	30.5
Cvetanovich et al. 2017 [73]	*Am J Sports Med*	Prospective	24.0	Knee	pACI, cACI	12	22.0%	17.0
			24.0	Knee	mACI	11	22.0%	17.0
			24.0	Knee	mACI	14	22.0%	17.0
D'Ambrosi et al. 2017 [74]	*Arthroscopy*	Retrospective	27.0	Talus	AMIC	17	52.9%	25.0
				Talus	AMIC	14	26.0%	47.0
D'Ambrosi et al. 2019 [75]	*Clin J Sport Med*	Retrospective	42.6	Talus	AMIC	26	34.6%	33.7
de l’Escalopier et al. 2015 [76]	*Orthop Traumatol Sur Res*	Retrospective	76.0	Talus	Mosaicplasty	37	33.0%	21.6
De Windt et al. 2017 [77]	*Stem Cells*	Prospective	12.0	Knee	Allo-MSC	10	20.0%	26.0
De Windt et al. 2017 [78]	*Stem Cells*	Prospective	18.0	Knee	Allo-MSC	35	31.0%	30.0
De Girolamo et al. 2019 [79]	*J Clin Med*	Prospective, randomized	100.0	Knee	AMIC	12	38.0%	30.0
				Knee	AMIC	12	50.0%	30.0
Desando et al. 2017 [80]	*Cartilage*	Prospective	36.0	Talus	MACI	7	42.9%	31.2
			36.0	Talus	MBMAC	15	33.3%	31.0
Dhollander et al. 2012 [81]	*Knee Surg Sports Traumatol Arthrosc*	Prospective	36.0	Knee	cACI	32	31.0%	30.0
Dixon et al. 2011 [82]	*Foot Ankle Int*	Retrospective	44.4	Talus	MACI	28	22.2%	41.0
Domayer et al. 2012 [83]	*Osteoarthritis Cartilage*	Retrospective	113.8	Talus	MFX	10	55.6%	30.8
			65.4	Talus	MACT	10	77.8%	25.4
Duramaz et al. 2018 [84]	*Knee Surg Sports Traumatol Arthrosc*	Retrospective	28.9	Talus	MFX	14	62.5%	34.6
				Talus	Control group			
Ebert et al. 2011 [85]	*Am J Sports Med*	Prospective	60.0	Knee	mACI	44	48.0%	39.0
Ebert et al. 2012 [86]	*Arthroscopy*	Prospective	24.0	Knee	mACI	20	50.0%	34.0
Ebert et al. 2015 [87]	*Am J Sports Med*	Prospective	24.0	Knee	mACI	10	20.0%	39.0
				Knee	mACI	13	7.0%	36.0
				Knee	mACI	9	66.0%	38.0
				Knee	mACI	15	53.0%	37.0
Ebert et al. 2017 [88]	*Am J Sports Med*	Prospective	60.0	Knee	mACI	31	51.0%	35.0
Efe et al. 2011 [89]	*Am J Sports Med*	Prospective	24.0	Knee	mACI	15	60.0%	26.0
El-Rashidy et al. 2011 [90]	*J Bone Joint Surg*	Retrospective	37.7	Talus	OAT	38	42.1%	44. 2
Emre et al. 2012 [91]	*J Foot Ankle Surg*	Retrospective	16.8	Talus	Mosaicplasty	32	9.4%	27.5
Enea et al. 2013 [92]	*Knee*	Retrospective	22.0	Knee	AMIC	9	45.0%	48.0
Enea et al. 2015 [93]	*Knee*	Retrospective	29.0	Knee	AMIC	9	44.0%	43.0
Espregueira-Mendes et al. 2012 [94]	*Knee Surg Sports Traumatol Arthrosc*	Prospective	110.0	Knee	OAT	31	29.0%	30.0
Ferruzzi et al. 2008 [95]	*J Bone Joint Surg*	Prospective	60.0	Knee	pACI	48	38.0%	32.0
				Knee	mACI	50	28.0%	31.0
Filardo et al. 2011 [96]	*Am J Sports Med*	Prospective	84.0	Knee	mACT	62	23.0%	28.0
Filardo et al. 2014 [26]	*BMC Musculos Dis*	Prospective	84.0	Knee	mACT	131	35.0%	29.0
Fraser et al. 2016 [97]	*Knee Surg Sports Traumatol Arthrosc*	Retrospective	70.8	Talus	OAT	36	3.3%	31.0
Galla et al. 2019 [98]	*Knee Surg Sports Traumatol Arthrosc*	Retrospective	33.5	Talus	AMIC	23	34.8%	35.6
Gaul et al. 2018 [99]	*Foot Ankle Int*	Retrospective	116.4	Talus	OAT	20	47.0%	34.7
Gaul et al. 2018 [100]	*Foot Ankle Int*	Retrospective	123.6	Talus	OAT	20	55.0%	43.6
Gautier et al. 2002 [101]	*J Bone Joint Surg*	Retrospective	24.0	Talus	OAT	11	66.5%	32.0
Georgiannos et al. 2016 [102]	*Knee Surg Sports Traumatol Arthrosc*	Retrospective	66.0	Talus	OAT	48	19.5%	36.0
Giannini et al. 2008 [103]	*Am J Sports Med*	Retrospective	36.0	Talus	mACI	46	37.0%	31.4
Giannini et al. 2009 [104]	*Am J Sports Med*	Retrospective	119.0	Talus	PACI	10	50.0%	25.8
Giannini et al. 2014 [105]	*Knee Surg Sports Traumatol Arthrosc*	Retrospective	87.2	Talus	mACI	46	36.9%	31.4
Giannini et al. 2017 [106]	*Injury*	Retrospective	121.0	Talus	OAT	48	25.0%	36.0
Gille et al. 2013 [107]	*Arch Orthop Trauma Surg*	Prognostic study	24.0	Knee	AMIC	57	33.0%	37.0
Giza et al. 2010 [108]	*Foot Ankle Int*	Retrospective	24.0	Talus	MACI	10	50.0%	40.2
Gobbi et al. 2006 [109]	*Arthroscopy*	Prospective	53.0	Talus	MFX	10	40.0%	24.0
				Talus	Control group			
				Talus	OAT	12	33.3%	27.8
Gobbi et al. 2009 [110]	*Am J Sports Med*	Prospective	60.0	Knee	mACI	34	32.0%	31.0
Gobbi et al. 2011 [111]	*Cartilage*	Prospective	24.0	Knee	cACI	15	33.0%	48.0
Gobbi et al. 2014 [112]	*Am J Sports Med*	Prospective	41.0	Knee	cACI	25	36.0%	47.0
Gobbi et al. 2017 [113]	*Knee Surg Sports Traumatol Arthrosc*	Prospective	48.0	Knee	cACI	20		50.0
				Knee	mACI	20		37.0
Gomoll et al. 2014 [114]	*Am J Sports Med*	Prospective	48.0	Knee	pACI	110	64.0%	33.0
Gooding et al. 2006 [115]	*Knee*	Prospective, randomized	24.0	Knee	pACI	33	51.0%	31.0
				Knee	cACI	35		
Gottschalk et al. 2017 [116]	*J Foot Ankle Surg*	Retrospective	60.0	Talus	AMIC	21	38.1%	37.0
Gudas et al. 2006 [117]	*Knee Surg Sports Traumatol Arthrosc*	Prospective, randomized	37.1	Knee	MFX	28	42.9%	24.3
				Knee	OAT	29	34.5%	24.6
Gudas et al. 2009 [118]	*J Pediatr Orthop*	Prospective, randomized	24.0	Knee	OAT	25	40.0%	15.0
				Knee	MFX	22	40.0%	14.0
Gudas et al. 2012 [119]	*Am J Sports Med*	Prospective, randomized	120.0	Knee	OAT	28	32.0%	25.0
				Knee	MFX	29	41.0%	24.0
Gudas et al. 2018 [120]	*J Orthop Surg*	Retrospective	54.0	Knee	AMIC	15	33.0%	31.0
Gül et al. 2016 [121]	*Foot Ankle Surg*	Retrospective	30.5	Talus	OAT	15	33.3%	32.6
			28.9	Talus	OAT	13	830.0%	36.7
Guney et al. 2016 [122]	*Knee Surg Sports Traumatol Arthrosc*	Prospective	47.3	Talus	MFX	19	37.4%	47.4
			40.4	Talus	MFX & PRP	22	43.9%	50.0
			30.1	Talus	Mosaicplasty	13	37.6%	15.4
Haleem et al. 2010 [123]	*Cartilage*	Retrospective	12.0	Knee	pACI	5	20.0%	25.0
Haleem et al. 2014 [124]	*Am J Sports Med*	Retrospective	93.0	Talus	OAT	14	50.0%	42.8
			85.3	Talus	OAT	28	39.3%	44.1
Haasper et al. 2007 [125]	*Arch Orthop Trauma Surg*	Retrospective	24.0	Talus	Mosaicplasty	14	57.1%	24.8
Hahn et al. 2010 [126]	*Foot Ankle Int*	Retrospective	47.9	Talus	OAT	13	61.5%	30.4
Hangody et al. 1997 [127]	*J Bone Joint Surg*	Retrospective	19.0	Talus	Mosaicplasty	11		25.1
Hangody et al. 2001 [128]	*Foot Ankle Int*	Retrospective	50.4	Talus	Mosaicplasty	36		27.0
Hoburg et al. 2019 [129]	*Orthop J Sports Med*	Prospective	63.0	Knee	mACI	29	48.0%	16.0
			48.0	Knee	mACI	42	29.0%	27.0
Horas et al. 2003 [130]	*J Bone Joint Surg*	Prospective	24.0	Knee	pACI	20	60.0%	31.4
				Knee	OAT	20	25.0%	35.4
Imhoff et al. 2011 [131]	*Am J Sports Med*	Retrospective	84.0	Talus	OAT	26	46.2%	33.0
Jackson et al. 2019 [132]	*Foot Ankle Surg*	Retrospective	21.0	Talus	OAT	31	9.7%	33.6
Kim et al. 2019 [133]	*Foot Ankle Int*	Retrospective	47.3	Talus	MFX	64	26.6%	40.5
Knutsen et al. 2016 [134]	*J Bone Joint Surg*	Prospective, randomized	180.0	Knee	pACI	40		
				Knee	MFX	40		
Koh et al. 2016 [135]	*Arthroscopy*	Prospective, randomized	27.0	Knee	MFX	40	65.0%	38.0
				Knee	MFX	40	60.0%	39.0
Kon et al. 2009 [136]	*Am J Sports Med*	Prospective	60.0	Knee	mACT	40	17.0%	29.0
				Knee	MFX	40	32.0%	31.0
Kon el al. 2011 [137]	*Am J Sports Med*	Prospective	61.0	Knee	mACT	22	32.0%	46.0
			58.0	Knee	mACI	39	35.0%	45.0
Kretzschmarr et al. 2015 [138]	*Eur Radiol*	Prospective		Talus	AMIC	25	32.0%	38.0
Kreulen et al. 2018 [139]	*Foot Ankle Spec*	Prospective	84.0	Talus	MACI	9	55.6%	45.8
Kreuz et al. 2005 [140]	*Am J Sports Med*	Retrospective	48.9	Talus	Mosaicplasty	35	48.6%	30.9
Kubosch et al. 2016 [33]	*Int Orthop*	Retrospective	39.5	Talus	AMIC	17	47.1%	38.8
Kwak et al. 2014 [141]	*Am J Sports Med*	Retrospective	70.0	Talus	PACI	29	48.3%	34.0
Lahner et al. 2018 [142]	*Biomed Res Int*	Prospective	14.7	Knee	AMIC	9		48.0
Lee et al. 2003 [143]	*Foot Ankle Int*	Retrospective	36.0	Talus	Mosaicplasty	18	5.6%	22.7
Li et al. 2017 [144]	*BMC Musculos Dis*	Retrospective	21.2	Talus	OAT	11	63.6%	55.4
Lim et al. 2012 [12]	*Clin Orthop Rel Res*	Prospective, randomized	60.0	Knee	MFX	30	40.0%	33.0
				Knee	OAT	22	45.0%	30.0
				Knee	pACI	18	44.0%	25.0
Liu et al. 2011 [145]	*Foot Ankle Int*	Prospective	36.3	Talus	OAT	16	37.5%	33.9
Liu et al. 2019 [146]	*Foot Ankle Soc*	Retrospective	18.0	Talus	OAT	14	21.4%	29.6
Lopez-Alcorocho et al. 2018 [147]	*Cartilage*	Prospective	24.0	Knee	mACI	50	30.0%	35.0
López-Alcorocho et al. 2019 [148]	*Cartilage*	Prospective	24.0	Talus	HD-ACI	26	41.7%	31.0
Macmull et al. 2011 [149]	*Int Orthop*	Prospective	66.0	Knee	pACI, cACI	24	29.0%	16.0
				Knee	mACI	7		
Macmull et al. 2012 [150]	*Am J Sports Med*	Prospective	45.0	Knee	cACI	25	80.0%	35.0
			35.3	Knee	mACI	23	61.0%	35.0
Magnan et al. 2012 [151]	*Advance Orthop*	Retrospective	45.0	Talus	MACI	30	50.0%	28.9
Marlovits et al. 2012 [152]	*Am J Sports Med*	Prospective	60.0	Knee	mACI	24	12.0%	35.0
McNickle et al. 2009 [153]	*Am J Sports Med*	Prospective	52.0	Knee	pACI	140	42.0%	30.0
Mehl et al. 2019 [154]	*Knee*	Retrospective	78.0	Knee	pACI, cACI	78	59.0%	32.0
Meyerkort et al. 2014 [155]	*Knee Surg Sports Traumatol Arthrosc*	Prospective	60.0	Knee	mACI	23		42.0
Micheli et al. 2001 [156]	*Clin J Sport Med*	Prospective	36.0	Knee	pACI	50	26.0%	36.0
Minas et al. 2014 [157]	*Clin Orthop Rel Res*	Prospective	120.0	Knee	pACI	210	46.0%	36.0
Moseley et al. 2010 [158]	*Am J Sports Med*	Prospective	110.0	Knee	pACI	72	39.0%	37.0
Murphy et al. 2019 [159]	*Knee Surg Sports Traumatol Arthrosc*	Retrospective	36.7	Talus	MAST	38	31.2%	35.0
Nam et al. 2009 [160]	*American Journal Sports Med*	Retrospective	37.5	Talus	PACI	11	54.5%	33.5
Nawaz et al. 2014 [161]	*J Bone Joint Surg*	Retrospective	74.0	Knee	pACI, cACI	827	40.0%	34.0
				Knee	mACI			
Nehrer et al. 2011 [162]	*Cartilage*	Prospective	61.0	Talus	pACT, MACT	17	58.8%	28.0
Nejadnik et al. 2010 [163]	*Am J Sports Med*	Retrospective	24.0	Knee	mACI	36	50.0%	43.0
				Knee	MFX	36	44.0%	44.0
Nguyen et al. 2019 [164]	*Am J Sports Med*	Retrospective	44.7	Talus	OAT	38	0.0%	26.0
Niemeyer et al. 2008 [165]	*Arch Orthop Trauma Surg*	Retrospective	38.0	Knee	pACI	95		34.0
				Knee	mACI			
Niemeyer et al. 2010 [166]	*Arthroscopy*	Prospective	37.0	Knee	cACI	59		37.0
Niemeyer et al. 2014 [167]	*Am J Sports Med*	Prospective	131.0	Knee	pACI	70	64.0%	33.0
Niemeyer et al. 2016 [168]	*Am J Sports Med*	Prospective, randomized	12.0	Knee	mACI	25	33.0%	33.0
				Knee	mACI	25	16.0%	34.0
				Knee	mACI	25	40.0%	34.0
Niemeyer et al. 2019 [169]	*Orthop J Sports Med*	Prospective, randomized	24.0	Knee	mACI	52	36.0%	36.0
				Knee	MFX	50	44.0%	37.0
Ogura et al. 2017 [170]	*Am J Sports Med*	Prospective	240.0	Knee	pACI	24	30.0%	35.0
Ogura et al. 2019 [171]	*Orthop J Sports Med*	Prospective	24.0	Knee	pACI, cACI	15	20.0%	31.0
Orr et al. 2017 [172]	*Foot Ankle Spec*	Retrospective	28.5	Talus	OAT	8	0.0%	34.4
Pagliazzi et al. 2018 [173]	*Foot Ankle Surg*	Retrospective	87.2	Talus	MACI	20	30.0%	35.0
Park et al. 2018 [174]	*American Journal Sports Med*	Retrospective	71.4	Talus	OAT	18	41.6%	
				Talus	OAT	28		
Park et al. 2020 [175]	*Bone Joint Journal*	Retrospective	22.0	Talus	OAT	25	40.0%	19.6
Paul et al. 2012 [176]	*Am J Sports Med*	Retrospective	60.0	Talus	OAT	131	38.2%	31.0
Peterson et al. 2010 [177]	*Am J Sports Med*	Retrospective	154.0	Knee	pACI	224		33.0
Polat et al. 2016 [178]	*Knee Surg Sports Traumatol Arthrosc*	Retrospective	121.3	Talus	MFX	82	41.5%	35.9
Quirbach et al. 2009 [179]	*Skeletal Radiol*	Retrospective	19.8	Talus	MACT	12	33.3%	32.8
Randsborg et al. 2016 [180]	*BMC Musculos Dis*	Prospective, randomized	24.0	Knee	cACI	82		
				Knee	Control group			
Richter et al. 2017 [181]	*Foot Ankle Surg*	Prospective	24.0	Talus	MAST	26	28.0%	33.0
Richter et al. 2020 [182]	*Foot Ankle Surg*	Prospective	24.4	Talus	MAST	129	41.0%	35.3
			23.8	Talus	AMIC	129	40.0%	35.6
Rosa et al. 2016 [183]	*J Orthop Traumatol*	Retrospective	148.0	Knee	pACI	15	40.0%	21.0
Ross et al. 2016 [184]	*Arthroscopy*	Retrospective	51.0	Talus	OAT	76	34.2%	35.8
Sabaghzadeh et al. 2019 [185]	*Chinese Journal of Traumatology*	Retrospective		Talus	Mosaicplasty	19	42.1%	43.0
Sadlik et al. 2016 [186]	*Foot Ankle Surg*	Retrospective	46.4	Talus	OAT	10	40.0%	37.0
Saris et al. 2009 [187]	*Am J Sports Med*	Prospective, randomized	36.0	Knee	pACI	57	39.0%	33.9
				Knee	MFX	61	33.0%	33.9
Saris et al. 2014 [188]	*Am J Sports Med*	Prospective, randomized	24.0	Knee	mACI	72	37.0%	35.0
				Knee	MFX	72		33.0
Schagemann et al. 2018 [189]	*Arch Orthop Trauma Surg*	Retrospective	24.0	Knee	AMIC	20	35.0%	38.0
				Knee	AMIC	30	43.0%	34.0
Schiavone Panni et al. 2018 [190]	*Knee Surg Sports Traumatol Arthrosc*	Retrospective	84.0	Knee	AMIC	21		
Schneider et al. 2009 [191]	*Foot Ankle Int*	Retrospective	21.1	Talus	MACI	20	65.0%	36.2
Schneider et al. 2011 [192]	*Am J Sports Med*	Prospective	30.2	Knee	mACI	116	42.0%	33.0
Schneider et al. 2016 [193]	*J Orthop Surg*	Prospective, Randomized	12.0	Knee	MFX	13	50.0%	47.0
				Knee	MFX	4		37.0
Schüttler et al. 2019 [194]	*Arch Orthop Trauma Surg*	Prospective	60.0	Knee	mACI	23	34.0%	
Siebold et al. 2018 [195]	*Knee Surg Sports Traumatol Arthrosc*	Prospective	34.8	Knee	mACI	30	36.0%	36.0
Shimozono et al. 2018 [196]	*Am J Sports Med*	Retrospective	52.0	Talus	OAT	63	42.9%	36.0
			45.0	Talus	OAT	31	32.3%	34.0
Shimozono et al. 2018 [197]	*Bone Joint Surg*	Retrospective	26.3	Talus	OAT	25	64.0%	38.4
			22.3	Talus	OAT	16	37.5%	43.6
Skowronski et al. 2013 [198]	*Orthop traumatol Rehab*	Prospective	60.0	Knee	cACI	21	42.0%	26.0
				Knee	cACI	25	44.0%	26.0
Solheim et al. 2018 [199]	*Am J Sports Med*	Prospective, randomized	180.0	Knee	MFX	20	30.0%	35.0
				Knee	Mosaicplasty	20	30.0%	31.0
Steinwachs et al. 2019 [200]	*Knee*	Retrospective	6.0	Knee	AMIC	93	28.0%	42.0
Teo et al. 2013 [201]	*Clin Orthop Rel Res*	Retrospective	24.0	Knee	pACI	20	20.0%	17.0
				Knee	pACI	3		
Tohyama et al. 2009 [202]	*J Orthop Sci*	Prospective	24.0	Knee	pACI	27		
Usuelli et al. 2016 [203]	*Knee Surg Sports Traumatol Arthrosc*	Retrospective	24.0	Talus	AMIC	20	45.0%	36.1
Valderrabano et al. 2013 [204]	*Am J Sports Med*	Retrospective	30.9	Talus	AMIC	26	30.8%	34.6
Van Assche et al. 2010 [205]	*Knee Surg Sports Traumatol Arthrosc*	Prospective, randomised	24.0	Knee	pACI	33	33.0%	31.0
				Knee	MFX	34	10.0%	31.0
Vanlauwe et al. 2011 [206]	*Am J Sports Med*	Prospective, randomised	60.0	Knee	MFX	61	20.0%	34.0
				Knee	pACI	51	43.0%	34.0
Vanlauwe et al. 2012 [207]	*Am J Sports Med*	Prospective	48.0	Knee	pACI	38	68.0%	31.0
Volz et al. 2017 [208]	*Int Orthop*	Prospective, randomised	60.0	Knee	AMIC	17	29.0%	34.0
				Knee	AMIC	17	11.0%	39.0
				Knee	MFX	13	23.0%	40.0
Von Keudell et al. 2017	*Cartilage*	Prospective	88.0	Knee	pACI	30		32.0
Weigelt et al. 2019 [34]	*Am J Sports Med*	Retrospective	56.4	Talus	AMIC	33	4.2%	35.1
Whittaker et al. 2005 [209]	*J Bone Joint Surg*	Prospective	23.0	Talus	PACI	10	30.0%	41.8
Wiewiorski et al. 2013 [210]	*Clin Radiology*	Retrospective	23.3	Talus	AMIC	23	30.4%	34.2
Wiewiorski et al. 2016 [211]	*Am J Sports Med*	Retrospective	46.9	Talus	AMIC	60	40.0%	34.9
Wolf et al. 2018 [212]	*Cartilage*	Prospective	24.0	Knee	MFX	18	55.0%	38.0
				Knee	MFX	3		50.0
Woelfle et al. 2013 [213]	*Knee Surg Sports Traumatol Arthrosc*	Retrospective	29.0	Talus	OAT	32	24.5%	46.9
Yontar et al. 2018 [214]	*Acta Orthop Traumatol Turc*	Retrospective	20.3	Talus	AMIC	20	30.0%	32.9
Yoon et al. 2014 [215]	*Am J Sports Med*	Retrospective	45.0	Talus	OAT	22	31.8%	37.1
		Retrospective	50.0	Talus	MFX	22	18.2%	41.6
Zaslav et al. 2009 [216]	*Am J Sports Med*	Prospective	48.0	Knee	pACI	154	31.0%	35.0
Zeifang et al. 2010 [217]	*Am J Sports Med*	Prospective, randomised	24.0	Knee	mACI	11	45.0%	29.0
				Knee	pACI	10	0.0%	30.0
Zhu et al. 2016 [218]	*Foot Ankle Soc*	Retrospective	25.4	Talus	OAT	12	38.5%	40.5

*Syn-MSC* synovial mesenchymal stem cell; *BMAC* bone marrow aspirate concentrate; *BM-MSC* bone marrow-derived mesenchymal stem cells; *Allo-MSC* allogenic mesenchymal stem cell

### Outcomes of interest

Female sex evidenced moderate association with greater VAS at last follow-up (*r* = 0.3; *P* = 0.02). Patient’s age evidenced negative association with the AOFAS score (*r* =  − 0.2; *P* = 0.04) and Lysholm Knee Scoring Scale (*r* =  − 0.4; *P* = 0.03). Greater BMI was moderately associated with the rate of graft hypertrophy (*r* = 0.6; *P* = 0.009). VAS, IKDC, AOFAS, and Tegner Activity Scale at baseline were positively associated with themselves at last follow-up: VAS (*r* = 0.9; *P* < 0.0001), IKDC (*r* = 0.5; *P* = 0.007), AOFAS (*r* = 0.6; *P* = 0.0002), Tegner Activity Scale (*r* = 0.4; *P* = 0.009). The VAS score at baseline was inversely associated with the Tegner Activity Scale (*r* =  − 0.8; *P* < 0.0001) at last follow-up. No further statically significant associations were evidenced. The results of each of the pairwise correlation is shown in greater detail in Table 2.

**Table 2:** Overall results of the multivariate analyses

Demographic data at baseline																				
Endpoint at last FU	Sex		Age		BMI		Defect size		Symptoms Duration		VAS		Tegner		Lysholm		AOFAS		IKDC	
	*r*	*P*	*r*	*P*	*r*	*P*	*r*	*P*	*r*	*P*	*r*	*P*	*r*	*P*	*r*	*P*	*r*	*P*	*r*	*P*
VAS	0.2	0.02	0.0	0.9	0.0	0.7	0.0	0.7	0.3	0.1	0.9	< 0.0001	0.3	0.2	− 0.3	0.2	− 0.3	0.2	0.1	0.7
Tegner	− 0.2	0.07	0.0	0.9	− 0.2	0.3	0.2	0.1	− 0.0	0.8	− 0.8	< 0.0001	0.4	0.009	0.0	0.9	− 0.0	0.9	− 0.1	0.5
AOFAS	0.1	0.4	− 0.2	0.04	0.0	0.7	0.0	0.9	− 0.1	0.7	− 0.1	0.4	− 0.1	0.9			0.6	0.0002		
Lysholm	− 0.3	0.05	− 0.3	0.03	0.3	0.2	0.0	0.7	0.2	0.6	− 0.1	0.5	− 0.4	0.05	0.2	0.1			0.3	0.3
IKDC	− 0.2	0.08	0.0	0.8	− 0.2	0.4	0.3	0.05	− 0.3	0.2	0.0	0.8	0.1	0.5	0.0	0.8			0.4	0.0007
Hypertrophy	0.2	0.2	0.0	0.8	0.6	0.009	− 0.1	0.4	0.1	0.5	− 0	0.1	0.0	0.9	0.1	0.7	0.1	0.7	0.0	0.9
Failure	0.0	0.5	0.0	1	0.2	0.2	0.0	0.5	0.0	0.6	− 0.0	0.9	− 0.2	0.3	− 0.2	0.4	− 0.2	0.4	− 0.0	0.9
Revision	0.0	0.5	0.0	0.8	0.2	0.2	0.1	0.1	0.3	0.1	0.2	0.2	0.0	0.9	− 0.4	0.1	− 0.4	0.1	− 0.2	0.4

Values of 0.1 <|$r$ |< 0.3, 0.3 <|$r$ |< 0.5, and |$r$ |> 0.5 were considered to have weak, moderate, and strong association, respectively

*FU* follow-up

## Discussion

The management of articular cartilage defects still presents a major challenge. Therefore, identification of prognostic factors would allow to predict the outcome of various surgical techniques in multiple joints, and it would help to educate patients on the success (or not) of their surgical intervention. Older age was associated with lower values of the AOFAS and Lysholm scores at last follow-up, while women evidenced a positive association with VAS. Given the weak associations between these endpoints, the role of sex and age still remain not fully defined. BMI evidenced a moderate positive association with the rate of graft hypertrophy. VAS, IKDC, AOFAS, and Tegner scores at baseline were associated among themselves at last follow-up, demonstrating that the final outcome is influenced by the pre-operative performance status of the patients. Interestingly, symptom duration prior to the surgical intervention and cartilage defect size did not show any significant association with the surgical outcome.

Neri et al. analyzed 48 patients who underwent microfractures of knee cartilage defects at a mean follow-up of 5.7 years [23]. Patients’ age, BMI, time from diagnosis to surgery, and size of the cartilage lesion were negatively associated with the functional outcome. Differences between these findings and our results may be explained by the fact that Neri et al. only included 48 patients treated for knee articular cartilage defects with a longer follow-up, while we included 8905 procedures including various treatment options with a variable follow-up. Similar findings were reported by Andriolo et al. in 113 patients with knee cartilage defects treated with matrix-assisted autologous chondrocyte implantation [24]. Older age, female sex, degenerative lesions, longer symptoms duration, and previous surgery were negatively associated with outcome.

Age has been identified as one of the most important factors for success in the treatment of cartilage defects [25, 26]. A study comparing microfracture to ACI or OATS showed better clinical outcomes for patients younger than 30 years compared to those older than 30 years, unrelated to treatment type [27, 28]. Robb et al. were able to identify age as a prognostic factor for lower clinical outcomes after treatment [29]. The structure and composition of the matrix molecules as well as the synthetic function of chondrocyte change with age, may explain the lower functional outcomes after cartilage defect management in older patients [30, 31]. These previous findings confirm our results of age being negatively associated with AOFAS and Lysholm scores.

Previously, it was also shown, in particular for the ankle joint, that patients’ BMI is a negative prognostic factor [32], with worse clinical outcomes for patients with a BMI > 30 kg/m^2^ [33, 34]. Jaiswal et al. found similar results showing an influence of BMI on the Modified Cincinnati Score after anterior cruciate ligament reconstruction and matrix-assisted autologous chondrocyte implantation [35]. The poorer results following cartilage repair in obese patients may be explained by an increase in mechanical forces across the joint leading to cartilage breakdown.

There was evidence of weak association between female sex and VAS. Females have lower femoral and retropatellar cartilage volumes than males, and this decreases with age [36]. These findings might explain the higher risk for knee osteoarthritis in women compared to men. Kreutz et al. studied 52 patients after ACI showing worse outcomes in women compared to men [37]. Furthermore, higher complication rates in cartilage repair surgery were found in women 24 months after surgery, which might be related to lower satisfaction levels in women, possibly resulting in more postoperative complaints [38–40].

Interestingly, cartilage defect size, in both the knee and ankle joints, is not associated with negative outcome. This was previously confirmed in studies highlighting that defect size does not predict the clinical outcome after treatment of cartilage defects, confirming that functional outcome seems to be independent of cartilage defect size [25, 41].

This study identified prognostic factors for successful cartilage repair management in the knee and ankle joints, regardless of the surgical procedure. However, there are also some limitations that need to be addressed. Although we followed established guidelines for the preparation of systematic reviews, the risk of bias of the included studies was only moderate, with acceptable methodological assessment. Given the lack of quantitative data, primary and revision settings could not be analyzed separately. To increase the pooling data, the sex of the patients, mean age and BMI, defect size, and mean length of prior symptoms duration were not analyzed separately according to the body location (knee and ankle). Furthermore, we considered only the most common surgeries strategy for chondral repair, potentially increasing the risk of selection bias. Given their uncertain results, less common or more innovative procedures were not considered. Given the lack of data, surgical indications were not considered separately for analysis. Patients with larger chondral and/ or osteochondral lesions (>5 cm^2^) and obese (BMI > 30 kg/m^2^) were not considered, as the surgical outcomes are strongly negatively influenced by these variables [33–35]. Large lesions require challenging surgery, with transplants and unpredictable outcome. Similarly, in obese patients, the articular cartilage is subjected to high loads, and lesions may not heal properly. Further clinical investigations are required to establish the proper management of chondral defects. Results from the present study should be considered in the light of these limitations. Further high-quality investigations should validate the results of the present study in a clinical setting.

## Conclusion

Our results suggest that the clinical outcomes were mostly related to the patients’ performance status prior surgery and that greater BMI could be associated with greater rate of hypertrophy. Female sex and older age evidenced fair influence on outcome, while symptom duration prior to the surgical intervention and cartilage defect size evidenced no association with the surgical outcome. These results should be interpreted in the light of the limitations of the present study, and further investigations are needed to validate them in a clinical setting.

## Supplementary Information

Below is the link to the electronic supplementary material.

Supplementary file1 (DOC 65 KB)

## Data Availability

The data underlying this article are available in the article and in its online supplementary material.

## Abbreviations

MFx: Microfracture
ACI: Autologous chondrocyte implantation
OAT: Osteoarticular auto- or allograft transplantation
AMIC: Autologous matrix-induced chondrogenesis
PROMs: Patient-reported outcome reports
VAS: Visual analogic scale
AOFAS: American Orthopedic Foot and Ankle Score
BMI: Body mass index
IKDC: International Knee Documentation Committee
FU: Follow-up
